# Association between Childhood Dental Experiences and Dental Fear among Dental, Psychology and Mathematics Undergraduates in Brazil

**DOI:** 10.3390/ijerph9124676

**Published:** 2012-12-17

**Authors:** Maurício A. Oliveira, Cristiane B. Bendo, Meire C. Ferreira, Saul M. Paiva, Miriam P. Vale, Júnia M. Serra-Negra

**Affiliations:** 1 Department of Pediatric Dentistry and Orthodontics, School of Dentistry, Universidade Federal de Minas Gerais, Pampulha Campus, Avenida Presidente Antônio Carlos, 6627, Belo Horizonte-MG, 31270-901, Brazil; E-Mails: mauricioliveira14@yahoo.com.br (M.A.O.); crysbendo@yahoo.com.br (C.B.B.); smpaiva@uol.com.br (S.M.P.); miriamodonto@gmail.com (M.P.V.); 2 Department of Dentistry, School of Biological and Health Sciences, Universidade Federal dos Vales do Jequitinhonha e Mucuri, Rua da Glória, 187, Diamantina-MG, 39100-000, Brazil; E-Mail: meirecofe@hotmail.com

**Keywords:** dental fear, dental phobias, epidemiology, pediatric dentistry, questionnaires, behavior, undergraduate student

## Abstract

The aim of the present study was to evaluate the association between childhood dental experiences and dental fear in adulthood among dentistry, psychology and mathematics undergraduate students. A cross-sectional study of 1,256 students from the city of Belo Horizonte, Brazil, was performed. Students responded to the Brazilian version of the Dental Fear Survey (DFS) and a questionnaire regarding previous dental experiences. Both the DFS and the questionnaire were self-administered. Association was tested using descriptive, bivariate and multivariate linear regression analysis, with a 5% significance level. Dentistry undergraduates reported lower scores than psychology (*p* < 0.001) and mathematics undergraduates (*p* < 0.05) for all three dimensions of the DFS. Negative dental experiences in childhood was associated with dimensions of Avoidance (B = 2.70, *p* < 0.001), Physiological arousal (B = 1.42, *p* < 0.001) and Fears of specific stimuli/situations (B = 3.44, *p* < 0.001). The reason for first visit to dentist was associated with dimensions of Physiological arousal (B = 0.76, *p* < 0.01) and Fears of specific stimuli/situations (B = 1.29, *p* < 0.01). Dentists should be encouraged to evaluate the dental fear of their patients before treatment. The DFS has been found to be an effective instrument for this purpose.

## 1. Introduction

Fear is a basic emotion, present in all ages, cultures, races and species, and is related to a real and external triggering stimulus that results in escape, fight or postponement behavior towards a threatening situation [1,2,3,4]. It is a defensive response to a perceived threat or the result of encountering the stimulus or situation presented in an environment reminiscent of the original fear experience. It may range from feelings of apprehension or discomfort to physical symptoms such as tachycardia, breathing difficulty, dizziness and sweating, caused by a sense of danger [2,3,4].

Dental fear is not an unusual condition, and may be a significant obstacle to effective dental care [3,5,6]. Painful, traumatic or negative dental experiences, especially in childhood, are important determinants of dental fear [1,7,8,9,10,11,12]. Individuals with high dental fear may delay dental visits or cancel appointments, resulting in a vicious cycle of dental fear characterized by avoidance, increased dental problems and symptomatic treatment needs, as well as feelings of guilt, embarrassment and inferiority [5,8,9,10,11,12,13,14,15]. These feelings are important elements for the maintenance of high levels of dental fear [14,15], and dental fear plays a crucial role in the development of this cycle. When there is deterioration of dental condition, both negative self-evaluation and cognitive perceptions of dental visits are affected [14,15].

Due to the importance of dental fear, several instruments have been developed to study behavior and attitudes towards dental care [2,7,16,17,18]. The Dental Fear Survey (DFS) is one such instrument which was developed among students, including undergraduates in the USA (Bellingham, WA) [7] and was validated among psychology undergraduates [17]. The DFS was cross-culturally adapted and validated among Brazilian psychology undergraduates [19] with higher DFS scores associated with longer intervals between dental visits. The DFS has been translated and validated for application in a range of different countries [10,16,20,21,22,23,24]. Furthermore, the DFS has been used to assess dental fear before dental treatment under sedation, and in the study of dental phobias [10,24]. However, in a search of the PubMed bibliographic database of the US National Library of Medicine, it was found that in Brazil only two studies with small convenience samples had applied the DFS [19,25]. 

Some studies of dental fear have been carried out among undergraduates [7,17,19,26,27]. Jordanian dental students reported lower dental anxiety than those from engineering and medical courses [26]. However, no Brazilian study investigating the prevalence of dental fear among undergraduates from different fields of study was found in extant literature. The aim of the present study was to measure dental fear among a large convenience sample in order to investigate: (1) if differences exist between different fields of study composed of undergraduates from health (dentistry), hard (mathematics) and soft (psychology) sciences and (2) if there is association between dental fear and negative dental experience in childhood.

## 2. Materials and Methods

### 2.1.Population and Study Design

The present cross-sectional study was conducted with dentistry, psychology and mathematics undergraduate students enrolled at the Universidade Federal de Minas Gerais (UFMG) from August to December 2010. UFMG is one of the most traditional and largest public universities in Brazil, offering 75 undergraduate and 247 postgraduate courses [28]. Students were residents of the city of Belo Horizonte, Minas Gerais, located in the south east of Brazil. All students enrolled in the three undergraduate courses were asked to participate in the study, from the first to the last year of each course, representing a total of 1,565 individuals. 

### 2.2. Data Collection

The participants were approached during lecture classes and asked to voluntarily participate in the study. Following authorization by the Human Research Ethics Committee of UFMG, and once a consent form had been signed, the participants self-completed the Brazilian version of the DFS, a pre-tested questionnaire used to collect socio-demographic data and information related to dental experience.

The DFS is a 20 item questionnaire relating to dental treatment, comprising three dimensions: Avoidance (eight items), Physiological arousal (five items) and Fears of specific stimuli/situations (seven items). The response options follow a rating scale ranging from “not at all” (score=1) to “very much” (score = 5). Avoidance scores can range from 8 to 40; Physiological arousal from 5 to 25; and Fears of specific stimuli/situations from 7 to 35. A higher score indicates greater dental fear [19].

During the first half of 2010, the questionnaires were administered in a pilot study of 80 students from the three courses. These students did not participate in the main study. The pilot study indicated that changes to the proposed methodology were not necessary. Intra-class correlation coefficient (ICC) for test-retest reliability of the DFS was obtained on two occasions, separated by an interval of two weeks. ICC results for mathematics, dentistry and psychology undergraduate students were 0.969 (95% CI: 0.945–0.986), 0.968 (95% CI: 0.953–0.980) and 0.949 (95% CI: 0.911–0.977), respectively. This data confirms the high degree of stability of the DFS.

### 2.3. Variables

Dental fear was the main outcome variable measured by the sum of items of each dimension of the DFS, and was used as continuous variable. For statistical analysis purposes, all categorical response variables were transformed into binary variables. Dummy variables were established for the “course of study” variable. Other independent variables, such as gender, age of first visit to the dentist, negative dental experiences in childhood and reason for first dental visit, were taken from the self-administered questionnaire. The variable “age of first childhood visit to the dentist” was dichotomized using the mean score as cut-point (≤6 years and >6 years). The variable “negative dental experience in childhood” was dichotomized into two alternatives: “yes” and “no”. In order to create the “yes” category, response options such as dental extractions, anesthetic needle, drill, extensive orthodontic treatment and inadequate behavior of dentist were combined. The “no” category corresponded to “no” responses in the questionnaire. The “reason for first dental visit” was dichotomized as routine examination and dental treatment (composed of toothache, fractures or dental caries). 

### 2.4. Statistical Methods

Data organization and statistical analysis was performed using the Statistical Package for the Social Sciences (SPSS for Windows, version 17.0, SPSS Inc., Chicago, IL, USA) program. Data analysis involved descriptive statistics, and the outcome was tested by bivariate and multivariate analysis. Chi-squared and One-Way ANOVA tests were also used for bivariate analyses. To avoid errors arising from multiple comparisons, the significance level was divided by the number of comparisons [29]. As the course of study variable was composed of three categories, it was necessary to perform multiple comparisons with Bonferroni corrections. The partition generated three multiple comparisons. P-values less than 0.017 were considered statistically significant in this case. The p-value was the result of 0.05 divided by 3 (0.05/3). Linear regression was conducted for bivariate analysis and multivariate models. The independent variables were introduced into the model, one by one, based on their statistical significance (*p* < 0.20) and/or epidemiological importance. The significance level was set at 5%.

## 3. Results

Of the total number of enrolled undergraduates, 505 students from dentistry, 442 from psychology and 309 from mathematics participated in the study. The response rate was 91.6% for dental students, 65.0% for psychology students and 92.5% for mathematics students. The main reasons for the non-response rate were the refusal of seven students to participate in the study and the absence of 302 students on data collection days. The highest rate of non-responses was from psychology undergraduates, as they did not attend lecture classes during the last two years of their course, making the application of the DFS and questionnaire more difficult in the case of these students. 

The age of the students ranged from 18 to 65 years with an average of 22.3 years (SD = 5.1). There was a predominance of females (62.9%) in the total population. However, the mathematics course had a higher proportion of male students (63.8%; *p* < 0.001) (Table 1).

Of those students who stated that they could recall their age at the time of their first dental visit (23.5%), the majority of dental (80.2%) and psychology (70.3%) undergraduates reported their first visit to the dentist as occurring by six years of age (*p* = 0.108; comparison between dentistry and psychology); while 54.2% of mathematics students reported their first dental visit as occurring after six years of age (*p* < 0.001; comparison between dentistry and mathematics; psychology and mathematics). Psychology students had a higher frequency of negative dental experience in childhood (30.4%; *p* < 0.001) compared to mathematics and dentistry students. Routine exam was the most reported reason for visits to the dentist in all three courses (76.5%), but mathematics undergraduates reported a higher demand for dental treatment (20.7%; *p* = 0.001) compared to dentistry students (Table 1).

**Table 1:** Frequency distribution and association of undergraduate students according to other independent variables.

Variables	Undergraduate Students n(%)				*p*-value	Partition with Bonferroni Correction
	Dentistry	Psychology	Mathematics	Total		
Gender (n = 1,256)						Dentistry *vs.* Psychology – *p* = 0.070
Male	156(30.9)	113(25.6)	197(63.8)	466(37.1)	<0.001 ^†^	Dentistry *vs* *.* Mathematics – *p* < 0.001
Female	349(69.1)	329(74.4)	112(36.2)	790(62.9)		Psychology *vs* *.* Mathematics – *p* < 0.001
Age (years) ^1 ^(n = 1,256)	21.6(3.7)	22.3(5.2)	23.2(6.5)	22.3(5.1)	<0.001 ^‡^	Dentistry *vs.* Psychology – *p* = 0.151
						Dentistry *vs* *.* Mathematics – *p* < 0.001
						Psychology *vs* *.* Mathematics – *p* = 0.043
Remember age of first dental visit ^2 ^(n = 1,254)						Dentistry *vs.* Psychology – *p* = 0.145
No	408(80.8)	340(76.9)	213(68.9)	961(76.5)	0.001 ^†^	Dentistry *vs* *.* Mathematics – *p* < 0.001
Yes	97(19.2)	102(23.1)	96(31.1)	295(23.5)		Psychology *vs* *.* Mathematics – *p* = 0.014
Age of first dental visit (n = 293)						Dentistry *vs.* Psychology – *p* = 0.108
≤6 years	77(80.2)	71(70.3)	44(45.8)	192(65.5))	<0.001 ^†^	Dentistry *vs* *.* Mathematics – *p* < 0.001
>6 years	19(19.8)	30(29.7)	52(54.2)	101(34.5		Psychology *vs* *.* Mathematics – *p* < 0.001
Negative dental experience in childhood ^2^ (n = 1,255)						Dentistry *vs.* Psychology – *p* < 0.001
No	411(81.4)	307(69.6)	243(78.6)	961(76.6)	<0.001 ^†^	Dentistry *vs* *.* Mathematics – *p* = 0.339
Yes	94(18.6)	134(30.4)	66(21.4)	294(23.4)		Psychology *vs* *.* Mathematics – *p* = 0.006
Reason for first dental visit ^2^						Dentistry *vs.* Psychology – *p* = 0.121
Routine examination (n = 935)	310(81.4)	256(76.2)	149(68.3)	715(76.5)	0.001 ^†^	Dentistry *vs* *.* Mathematics – *p* < 0.001
Dental treatment	71 (18.6)	78 (22.8)	69 (31.7)	220 (23.5)		Psychology *vs* *.* Mathematics – *p* = 0.031

Notes: n = number; % = percent. **^†^** Chi-square test; ^‡^ One-Way ANOVA; Bonferroni corrections (*p* < 0.017). ^1 ^The variable “age” was presented as mean and standard deviation (in parentheses) values. ^2^Not all students answered all questions.

**Table 2:** Bivariate linear regression for each dimension of DFS according to independent variables.

Variables	Avoidance			Physiological Arousal			Fears of Specific Stimuli/Situations		
	Mean (SE)	B	*p*-value	Mean (SE)	B	*p*-value	Mean (SE)	B	*p*-value
Course of study									
Dentistry	9.85 (0.13)	Ref.		7.24 (0.11)	Ref.		12.52 (0.23)	Ref.	
Psychology	12.43 (0.26)	2.58	<0.001	8.86 (0.18)	1.62	<0.001	18.87 (0.33)	6.34	<0.001
Mathematics	11.57 (0.27)	1.72	<0.001	8.02 (0.18)	0.78	0.001	16.14 (0.40)	3.61	<0.001
Gender									
Male	10.94 (0.21)	Ref.	0.152	7.79 (0.14)	Ref.	0.075	14.72 (0.31)	Ref.	<0.001
Female	11.32 (0.16)	0.38		8.12 (0.12)	0.33		16.19 (0.25)	1.48	
									
Negative dental experience during childhood									
No									
Yes	10.45 (0.12)	Ref.	<0.001	7.58 (0.09)	Ref.	<0.001	14.58 (0.20)	Ref.	<0.001
	13.56 (0.35)	3.10		9.36 (0.23)	1.78		19.13 (0.44)	4.55	
Age of first dental visit									
≤6 years	11.30 (0.36)	Ref.	0.993	8.05 (0.24)	Ref.	0.143	15.73 (0.52)	Ref.	0.269
>6 years	11.31 (0.43)	0.01		8.65 (0.33)	0.60		16.68 (0.66)	0.957	
Reason for first dental visit									
Routine examination	10.75 (0.15)	Ref.	<0.001	7.68 (0.11)	Ref.	<0.001	15.07 (0.25)	Ref.	<0.001
Dental treatment	12.11 (0.34)	1.36		8.71 (0.23)	1.03		17.25 (0.48)	2.17	

Notes: n = number, *p*-value = probability value; r = correlation coefficient; SE = standard error; B = unstandardized coefficient. Values in parentheses refer to Standard Errors (SE); Bivariate linear regression at a 5% level.

**Table 3:** Multivariate linear regression models for each dimension of DFS according to independent variables (n = 934).

Variables	Avoidance				Physiological Arousal				Fears of Specific Stimuli/Situations			
	B	SE B	β	*p*-value	B	SE B	β	*p*-value	B	SE B	β	*p*-value
Intercept	8,89	0.50			6.80	0.16			11.70	0.33		
Course of study												
Dentistry	Ref.				Ref.				Ref.			
Psychology	2.33	0.64	0.25	<0.001	1.40	0.22	0.22	<0.001	5.82	0.46	0.41	<0.001
Mathematics	1.74	0.66	0.18	0.009	0.51	0.25	0.07	0.045	3.09	0.52	0.19	<0.001
Negative dental experience during childhood												
No	Ref.			<0.001	Ref.			<0.001	Ref.			<0.001
Yes	2.70	0.62	0.26		1.42	0.24	0.19		3.44	0.49	0.21	
Reason for first dental visit												
Routine examination	Ref.			0.136	Ref.			0.001	Ref.			0.007
Dental treatment	0.86	0.57	0.09		0.76	0.23	0.10		1.29	0.48	0.08	

R^2^ = 15.1%, df = 4, *p* < 0.001 (Avoidance); R^2^ = 10.0%, df = 4, *p* < 0.001 (Physiological arousal); R^2^ =21.3%, df =4, *p* < 0.001 (Fears of specific stimuli/situations). Notes: n = number; B = unstandardized coefficient; SE B = standard error of B; β = standardized coefficient; df = degrees of freedom; *p*-value = probability value. Multivariate linear regression at a 5% level. Model adjusted for gender and age of first dental visit.

Bivariate linear regression showed that course of study, negative dental experience during childhood and reason for the first dental visit were associated with the Avoidance, Physiological arousal and Fears of specific stimuli/situations dimensions (Table 2).

Gender was significantly associated with only the Fears of specific stimuli/situations dimension. Females had a 1.48 times greater score in the Fears of specific stimuli/situations dimension than males (Table 2).

The results of multivariate linear regression analysis are shown in Table 3. The model was adjusted for gender and age of first dental visit. The final model presents unstandardized coefficients (B), standard errors of B (SE B) and standardized coefficients (β) explaining the association of the independent variables on the outcome variables (dental fear in each DFS dimension). The three DFS dimensions were associated with course of study and negative dental experience in childhood. Dentistry undergraduates reported lower scores than psychology (*p* < 0.001) and mathematics undergraduates (*p* < 0.05) for all three dimensions of the DFS. The B coefficients indicated that having had a negative dental experience in childhood (change from “no” to “yes”) resulted in an increase of 2.70 in the Avoidance dimension, 1.42 in the Physiological arousal dimension and 3.44 in the Fears of specific stimuli/situations dimension (*p* < 0.001). The final model showed that the reason for the first visit to dentist was associated with the Physiological arousal (B = 0.76; *p* < 0.01) and Fears of specific stimuli/situations (B = 1.29; *p* < 0.01) dimensions. 

## 4. Discussion

The present cross-sectional study showed that dental fear differed significantly among psychology, mathematics and dentistry undergraduates. Mathematics and psychology undergraduates had higher scores of dental fear than dentistry undergraduates in the DFS dimensions “Avoidance, Psychological arousal and Fear of specific stimuli/situations”, perhaps because of a lack of knowledge of the dental treatment which they had received [6,26]. The Fear of specific stimuli/situations dimension was comprised of items relating to operative procedures, and dentistry students scored lower in this specific dimension than undergraduates from the other courses of study. Further studies are necessary to better understand differences in the Avoidance and Psychological arousal dimensions. As fear is a complex emotion [2,13,30], the personality traits of the participants should be taken into consideration. Although dental fear was lower among dental students compared with others, they still reported some fear in all the three dimensions. Therefore, it is necessary to consider the future of dental professionals who are afraid of the procedures they perform on their patients [27]. 

Although data collection was thoroughly planned to include all undergraduates of dentistry, psychology and mathematics, it was verified that 19.75% of the asked students did not participate in the study. The non-response rate was distributed in an unequal fashion. The majority of non-respondents were from the psychology course, as they did not attend lecture classes in the final years of the course, making it difficult to apply the instruments. This may compromise the ability to generalize the results obtained from the psychology sub-sample to the original sample. 

The vast majority of undergraduates did not remember the age of their first visit. Although questionnaires are the main instruments for collecting data in surveys [31], the recall method based on data collected through retrospective questionnaires may influence the respondents, resulting in recall errors [31,32]. Recall error is an inaccuracy of recall, because of an insufficient ability to acquire, process and recall information [33]. The result of the present study should be considered with caution, as the recall method was used to investigate dental experience in childhood. However, approximately three-fourth of respondents claimed to remember the reason for their first dental visit. Routine exam was the most frequent reason for the first visit to the dentist reported by students from all three undergraduate courses, followed by operative dental treatments, which represent more preventive behavior on the part of the parents/guardians of these individuals when they were children. This result indicates a paradigm shift in health care promotion, compared to a study performed during the period when the DFS was first validated for use in Brazil. This previous study was conducted with undergraduates in the early 1990s [19], in which most students reported having visited the dentist for the first time for operative dental treatment (51.6%), and a minority reported visiting the dentist for routine examinations (23.0%). A pattern of regular dental visits for routine examinations during childhood could result in an absence of dental fear in adulthood. Moreover, expressions of fear behavior observed during dental appointments should be addressed by the dentist using cognitive and behavioral strategies, promoting appropriate management of the child.

The results of the present study were in agreement with previous research where undergraduates who had a negative dental experience in childhood had significantly higher scores of dental fear than those who reported no such experience [3,7,8,9]. The model adjusted for course of study and negative dental experiences in childhood demonstrated that undergraduates who visited the dentist for the first time due to operative dental treatment had significantly higher scores in the Psychological arousal and Fears of specific stimuli/situations dimensions than those who underwent routine examinations. These results confirm a significant association between routine dental examination in childhood and low dental fear in adulthood, as reported in a previous study [30]. Although the origins of fear towards dental treatment are many and complex [2,3] the present study is consistent with literature related to the subject which found that negative dental experience in childhood could significantly predict the persistence of high dental fear in adults [1,9,10,11]. 

Most previous studies reported that females had a higher prevalence of dental fear than males [6,9,10,11,13,20,22]. The present study demonstrated that females had higher scores for the Fears of specific stimuli/situation dimension than males; however, this difference did not remain significant when included in the adjusted multivariate model. There was no association between gender and the other two dimensions. The validation study of the DFS for Brazilian Portuguese language did not report a difference in DFS scores between males and females [19]. This previous Brazilian study used the total score of DFS, and the present study analyzed each of the three dimensions separately, which may explain the divergence in results.

Evaluation of fear recognition in adults using a questionnaire relating to first dental visit may allow a more suitable approach to patient care [9,16,22]. The DFS is a self-administered questionnaire for the surgery waiting room and is suitable for assessing fear of specific dental treatment items [22]. The well-informed dentist could establish a relationship of trust with the patient, allowing more effective dental care [9,11,16]. However, the information bias in studies using self-assessment questionnaires should be taken into account, since the quality of data could be influenced by this type of bias, so compromising the scientific results [32,34]. Dental fear is a common problem in dental treatment, and it is important for dental schools to encourage the study of behavioral sciences, with a focus on the patient-dentist relationship [27]. The dentist should be conscious of an appropriate way of dealing with the fear of children and minimizing the consequences of negative dental experience in childhood [9,11,13,16,17,20]. 

## 5. Conclusions

The results showed that dentistry undergraduates had a lower level of dental fear than either psychology or mathematics students. Additionally, undergraduates who reported a negative dental experience during childhood and had visited the dentist for the first time for operative dental treatment had a higher level of dental fear than those who had not suffered negative dental experiences during childhood. Therefore, dentists should be encouraged to measure the dental fear of patients before treatment, and the DFS has been found to be an effective instrument for this purpose. Furthermore, appropriate conduct of pediatric dentists is extremely important during the treatment of children in situations that can lead to fear, and should include cognitive and behavioral strategies that allow a more suitable approach to the treatment of children, so helping to avoid dental fear in adulthood and contributing to improvements in health care promotion.
